# Association between Urinary Polycyclic Aromatic Hydrocarbon Metabolites and Sperm DNA Damage: A Population Study in Chongqing, China

**DOI:** 10.1289/ehp.1002340

**Published:** 2010-12-09

**Authors:** Xue Han, Niya Zhou, Zhihong Cui, Mingfu Ma, Lianbing Li, Min Cai, Yafei Li, Hui Lin, Ying Li, Lin Ao, Jinyi Liu, Jia Cao

**Affiliations:** 1 Department of Hygiene Toxicology, College of Preventive Medicine, Third Military Medical University, Chongqing, People’s Republic of China; 2 Chongqing Family Planning Research Institute, Chongqing, People’s Republic of China; 3 Department of Epidemiology, College of Preventive Medicine, Third Military Medical University, Chongqing, People’s Republic of China

**Keywords:** DNA damage, environmental exposure, human, male, metabolites, polycyclic aromatic hydrocarbon, reproduction, semen, sperm

## Abstract

**Background:**

Polycyclic aromatic hydrocarbons (PAHs), a class of the most ubiquitous environmental contaminants, may reduce male reproductive functions, but the data from human population studies are very limited.

**Objectives:**

We designed this study to determine whether environmental exposure to PAHs contributes to the alteration in semen quality, sperm DNA damage, and apoptosis in the general male human population.

**Methods:**

We measured urinary levels of four PAH metabolites and assessed semen quality, sperm apoptotic markers with Annexin V assay, and sperm DNA damage with comet assay in 232 men from Chongqing, China.

**Results:**

We found that increased urinary 2-hydroxynaphthalene (2-OHNa) levels were associated with increased comet parameters, including the percentage of DNA in the tail (tail%) [β coefficient = 13.26% per log unit 2-OHNa (micrograms per gram creatinine); 95% confidence interval (CI), 7.97–18.55]; tail length (12.25; 95% CI, 0.01–24.52), and tail distribution (7.55; 95% CI, 1.28–18.83). The urinary level of 1-hydroxypyrene was associated only with increased tail% (5.32; 95% CI, 0.47–10.17). Additionally, the increased levels of four urinary PAH metabolites were significantly associated with decreased vital Annexin V negative sperm counts. However, there was no significant association between urinary PAH metabolite levels and human semen parameters or morphology of the sperm samples.

**Conclusions:**

Our data indicate that the environmental level of PAH exposure is associated with increased sperm DNA damage but not with semen quality. These findings suggest that exposure to PAHs may disrupt sperm DNA and thereby interfere with human male fertility.

Polycyclic aromatic hydrocarbons (PAHs) are a large family of environmental pollutants generated from the incomplete combustion of coal, wood, oil, gas, garbage, or other organic substances. PAH exposure is a major public health concern worldwide, because the active metabolites of PAHs are believed to act as mutagens and carcinogens and because PAHs are associated with an increased risk for developing many types of cancers, including lung, prostate, skin, lymphatic, and hematopoietic malignancies [Armstrong et al. 2004; Agency for Toxic Substances and Disease Registry (ATSDR) 1995; Rybicki et al. 2008].

Accumulating evidence has also suggested that PAH exposure has harmful effects on reproductive health. Early studies demonstrate that intraperitoneal injection of benzo(*a*)pyrene into adult rats resulted in the atrophy of seminiferous tubules and a lack of spermatids and spermatozoa (Payne 1958). *In utero* exposure to benzo(*a*)pyrene (10 mg/kg body weight) markedly impairs the fertility of F1 male mice (MacKenzie and Angevine 1981). Moreover, subacute exposure of male rats to inhaled benzo(*a*)pyrene leads to reduced testosterone concentrations and impaired epididymal function (Inyang et al. 2003; Ramesh et al. 2008). More recent reports have also suggested that certain PAHs or their metabolites can interact with the estrogen receptor (ER) and initiate ER signaling pathways *in vitro* and *in vivo* (Kummera et al. 2008; Vinggaard et al. 2000).

Similar effects for PAHs on the male reproductive system have been reported among infertility clinic clients or occupational populations. For example, a recent study (Xia et al. 2009) reported that exposure to PAHs at environmental levels is associated with an increased risk of male idiopathic infertility. Hsu et al. (2006) reported that the ambient PAH levels of occupationally exposed individuals are associated with decreased spermatozoa quality. However, the biological effects of PAHs on the reproductive system have not been confirmed in the general population.

Our study was designed to investigate the relationships between PAH exposure and male reproductive function in a general population in the city of Chongqing, China. Chongqing is one of the largest cities with heavy industries along the Yangtze River. In our earlier study, we found that PAHs were some of the most ubiquitous environmental contaminants in the Chongqing of southwestern China and that the levels of PAHs in this city were higher than those in other cities (Guo et al. 2006; Shu et al. 2002; Tian et al. 2003). In another study, Li et al. (2009) found that 61.1% of male subjects from the general population in Chongqing had one or more semen parameters below those recommended by the World Health Organization (WHO 1999). Based on these observations, we employed the hydroxylated metabolites of PAH present in human urine samples, including 2-hydroxynaphthalene (2-OHNa), 9-hydroxyphenanthrene (9-OHPh), 2-hydroxyfluorene (2-OHFlu), and 1-hydroxypyrene (1-OHP) as biomarkers of PAH exposures, and we evaluated the potential association between exposures to PAH and semen quality and sperm genetic integrity.

## Materials and Methods

### Study population

The men included in this study were subjects of an ongoing cross-sectional study of semen quality in the general population in Chongqing in 2007 (Li et al. 2009). We worked with Chongqing Family Planning Research Institute and its network of clinics to recruit volunteers. The trained staff informed the men about the purpose of the study and explained that no risks or discomfort would arise from participation in the research. Participants could stop participating in the study at any time for any reason. A questionnaire, physical examination, and semen collection were carried out at the reproductive unit at Chongqing Family Planning Research Institute. All the men were required to be permanent residents of the Chongqing area, 20–40 years of age, and without reproductive or urological diseases or occupational exposure to PAHs (e.g., road paving, coke oven work, aluminum industry, and bitumen manufacturing). The participants were instructed to abstain from ejaculation for 2–7 days before producing the semen samples. Written informed consent was obtained from all participants. The study proposal was reviewed and approved by the Ethical Committee of the Third Military Medical University.

Of the 1,346 subjects included in our semen quality study during the period from June to December 2007, we only included those who participated during the winter (i.e., December) in the present study (*n* = 232), because the winter weather may have a slight influence on sperm quality (Carlsen et al. 2004; Chen et al. 2003; Levine et al. 1990) and the level of PAH pollution is relatively heavy and stable during this time [Chongqing Environmental Protection Bureau (CEPB) 2007]. There were no statistical differences between the subjects included in this study and the rest of the study population with regard to demographic characteristics and socioeconomic status.

### PAH metabolites analysis

Urine specimens and semen samples were collected on the same day. Urine samples were stored at −20°C and kept away from light until analysis. Four hydroxylated PAH metabolites (2-OHNa, 9-OHPh, 2-OHFlu, and 1-OHP) in the urine samples were analyzed by an experienced analytical chemistry technician in a blind fashion.

The details of the analytical procedure have been described previously (Wang et al. 2005). In brief, urine samples (8.0 mL) were combined with hydrochloric acid (HCl) to adjust the pH to 5.0, followed by hydrolysis with β-glucuronidase and arylsulfatase (HP-2; Sigma-Aldrich, Inc., St Louis, MO, USA) in sodium acetate buffer. The mixture was shaken overnight at 37°C. After centrifugation, the hydrolyzed urine samples were extracted using solid phase extraction (SPE) cartridges (500 mg/mL; Supelco, Inc., Bellefonte, PA, USA) at a flow rate of < 1 mL/min. The extracts were concentrated under a stream of nitrogen gas, then analyzed by high-performance liquid chromatography with a fluorescence detector (HPLC-FD, Agilent 1100 series and Agilent Technologies 1200 series; Agilent, Inc., Palo Alto, CA, USA). Creatinine, which was used to adjust PAH concentrations, was measured in all samples by an automated chemistry analyzer (Shimazu CL-8000; Shimazu, Inc., Tokyo, Japan).

Samples containing creatinine concentrations > 3.0 or < 0.3 g/L were excluded, as these sample concentrations were too high or too low to get valid results (Xia et al. 2009). The lower limit of detection was 0.12 μg/L for 1-OHP, 0.37 μg/L for 2-OHNa, 0.64 μg/L for 9-OHPh, and 0.68 μg/L for 2-OHFlu. The intraday variations ranged from 1.92% for 2-OHNa to 3.68% for 1-OHP, and the interday variations ranged from 4.43% for 1-OHP to 9.87% for 2-OHFlu. The SPE recoveries ranged from 79.8% to 106.3%.

### Semen analysis

Semen samples were donated by masturbation after a self-reported abstinence period of 2–7 days. Samples were allowed to liquefy by incubation at 37°C in a water bath; almost all samples were liquefied within 1 hr. We used 1-milliliter samples to assess apoptosis and seminal parameters; the rest of the sample was kept in liquid nitrogen (−196°C) in small aliquots for the comet assay (Donnelly et al. 2001).

Two well-trained technicians performed routine semen analyses, including sperm volume (milliliters), concentration (×10^6^/mL), sperm count, pH value, progressive motile spermatozoa (grade [A + B]%), and rapid motile spermatozoa (grade A%) in a blind fashion according to the WHO guidelines (WHO 1999). Sperm volume was measured by aspiration into a 10-mL pipette, providing 0.1-mL accuracy. Sperm concentration was determined using a micro cell as a counting chamber. Sperm motility was assessed at 20× magnification on the heating stage of a microscope (37°C), and spermatozoa were scored in categories A, B, C, and D. All the samples were analyzed within 60 min of collection. For the assessment of sperm morphology, two fresh semen smears were made and stained using the method described in the 1999 WHO manual. At least 200 spermatozoa were counted and categorized as normal or abnormal on the basis of their morphology.

To reduce the variation in assessment of sperm characteristics, all analyses of semen quality were performed by the same two technicians. The technicians at the reproductive unit at Chongqing Family Planning Research Institute were well trained in semen analysis and participated in the Continuous Quality Control System (an external quality control system established on the basis of WHO guidelines).

### Annexin V assay

One of the earliest apoptotic events occurs when the membrane phospholipid phosphatidylserine (PS) translocates to the outer leaflet of the plasma membrane (Vermes et al. 1995). The membrane PS translocation of sperm was determined by Annexin V staining in combination with the propidium iodide (PI), which stains dead cells, according to the manufacturer’s instructions (Bender MedSystems, Vienna, Austria). Briefly, 0.5-mL semen samples were washed with phosphate-buffered saline (PBS), and the sperm were then resuspended in the binding buffer to obtain a cell density of approximately 5 × 10^5^. A 50-μL semen specimen plus 5 μL Annexin V–FITC were added to 145 μL buffer, and the mixture was then incubated at room temperature for 10–15 min. Samples were then washed once with buffer and resuspended in 200 μL buffer containing 1 μg/mL of PI. Samples were immediately analyzed by flow cytometry (Becton Dickinson, San José, CA, USA). All tests were run in duplicate. Sperm were classified as Annexin V^−^/PI^−^ spermatozoa (living cells without PS translocations), Annexin V^+^/PI^−^ spermatozoa (living cells with PS translocations), or PI^+^ spermatozoa (necrotic cells), and the results were expressed as the percentage of total sperm.

### Comet assay

The DNA integrity of individual spermatozoa was determined using the alkaline single-cell gel electrophoresis assay (i.e., comet assay). The procedure was based on existing methods (Irvine et al. 2000) with slight modifications. Briefly, 10-μL sperm samples were embedded in low melting point agarose (Sigma-Aldrich) and added to a slide covered with normal agarose. Slides were then immersed in cold lysis buffer solution [2.5 M sodium chloride (NaCl), 100 mM EDTA, 10 mM Tris (hydroxymethyl) aminomethane hydrochloride, 10% dimethylsulfoxide (DMSO), and 1% Triton X-100, pH 10.0] for 1 hr to dissolve the membranes and break down the protein matrices. After lysis, slides were incubated overnight at 37°C in a solution of proteinase K (100 mg/mL proteinase K in 2.5 M NaCl, 100 mM EDTA, 10% DMSO, pH 7.40). Slides were then transferred to an electrophoretic system with alkaline buffer (300 mM sodium hydroxide, 1 mM EDTA, pH 12.0) for 1 hr to allow DNA unwinding. The slides were washed twice with neutralization buffer, followed by staining with 20 μg/mL ethidium bromide, and observed under a fluorescence microscope (Eclipse E2000-S; Nikon, Inc., Tokyo, Japan). For each sample, two duplicate slides were prepared, and 100 randomly selected cells were scored for each slide. The percentage of tail DNA (tail%), tail length, and the tail distributed moment (TDM) were evaluated with the Comet Assay Software Project Lab (CaspLab 2004) image analysis system.

### Statistical analysis

Bivariate associations between each of the metabolites and each of the semen parameters were evaluated by Spearman correlation coefficient analyses. According to the previously reported method (Duty et al. 2003a), semen parameters were dichotomized based on the WHO reference values for sperm number (≥ 40 × 1 0^6^), sperm motility (grade A + B ≥ 50% or grade A ≥ 25%), and normal morphology (≥ 15%) (Luben et al. 2007). The men with all three semen parameters greater than or equal to the reference values were defined as the comparison group; a man could contribute data to any or all of the below-referenced value groups. The relationships between the dichotomized semen parameters and categorized (tertiles and quartiles) PAH metabolite concentrations were analyzed using nonconditional logistic regression models. Linear regression analysis was also used to explore the association between continuous measures of semen quality and urinary PAH metabolites.

The associations between spermatozoa damage (comet assay and apoptosis parameters) and continuous measures of urinary PAH metabolites were analyzed using multivariate linear regression analysis. Because the distributions of PAH metabolite levels and the percentages of Annexin V^+^/PI^−^ were skewed, log transformation was applied for the analyses. The percentage of Annexin V^−^/PI^−^ spermatozoa, tail%, tail length, and TDM were close to normal distribution and therefore used without transformation in the analyses.

As potential confounders, age, body mass index (BMI), duration of abstinence (as continuous), smoking status (no smoking, ≤ 10 and > 10 cigarettes/day), alcohol consumption (no drinking, ≤ 120-g standard drinks/month, and > 120-g standard drinks/month), and grilled and smoked foods ingestion (grams per week) were included in the analysis. We used the change-in-estimate method (Greenland 1989) to decide which of the potential confounders to adjust for in the multivariate models. Potential confounders were included if the regression coefficient was changed by more than 10% when they were included one by one in the multivariate models. The Statistical Package for the Social Sciences (SPSS) version 13.0 (SPSS Inc. Chicago, Illinois, USA) was used for statistical analysis.

## Results

### Participants

All of the 232 participants were Han Chinese with an average age of 32 years, mean BMI of 22.55 kg/m^2^, and an average abstinence period of 4.63 days (range from 2 to 7 days). The participants were asked to report their smoking and alcohol intake during the 6 months prior to sample collection. As shown in Table 1, more than half of the participants used tobacco (62.5%) and alcohol (67.7%). The Annexin V/PI assay was not performed on the samples from 10 men, and the sperm samples from 11 men were not archived for the comet assay because of limited semen volume (< 1.2 mL). In addition, eight semen samples were lost during the comet assay.

### PAH metabolites

The creatinine-adjusted concentrations for 2-OHNa, 9-OHPh, 2-OHFlu, and 1-OHP are presented in Table 2. The detection rate of the four PAH metabolites was 100%. The urinary creatinine level for all samples was within the acceptable range (0.27–3.10 g/L). 2-OHNa had the highest geometric mean (GM), followed by 2-OHFlu, 9-OHPh, and 1-OHP.

### Male reproductive characteristics

Of the 232 semen samples evaluated, 139 (59.91%) had at least one semen parameter (volume, concentration, count, pH, sperm motility, or percentage of normal sperm) below the WHO reference values (WHO 1999), with 71 (30.60%) having sperm motility values less than the reference values and 30 (12.93%) having abnormal sperm morphology. There was no significant difference in the normal rate between the subjects included in this study (*n* = 232, 40.09%) and the rest of the greater study population (*n* = 1,114, 39.67% reached all the WHO reference values).

### PAH exposure and semen parameters

In the preliminary nonparametric correlation analysis, 2-OHFlu showed a weak negative correlation with sperm count (Spearman coefficient = 0.15, *p* = 0.03). However, when adjusted for duration of abstinence, which is associated with sperm count, this association was no longer significant (*p* > 0.05). There were no significant correlations between 9-OHPh or 1-OHP and sperm count, sperm concentration, or sperm motility. The Spearman coefficients between the levels of 2-OHNa and semen parameters were near zero and not significant.

The results from logistic regression models were similar to the linear regression models, that is, there were no statistically significant associations between PAH metabolites and semen parameters (data not shown).

### PAH exposure and sperm qualities

The final multiple regression models are summarized in Table 3. Although the coefficients did not differ by more than 10% with adjustment for age, we included age in the final model, because other studies have reported that age is a predictor of spermatic DNA damage and apoptosis (Wyrobek et al. 2006).

There were negative associations between the percentage of Annexin V^−^/PI^−^ spermatozoa and the concentrations of 2-OHNa, 2-OHFlu, 9-OHPh, and 1-OHP. The percentages of PI^+^ spermatozoa were positively associated with the levels of PAH metabolites. Coefficients for the relationship between the percentages of Annexin V^+^/PI^−^ spermatozoa and PAH metabolites were near zero and not significant.

**Figure 1:**
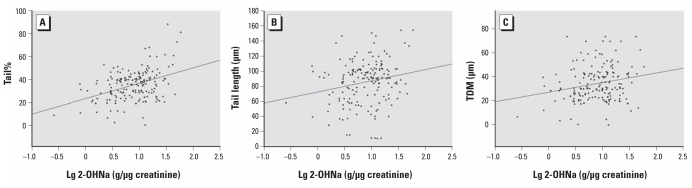
The crude association between the log of the urinary concentration of 2–OHNa and the comet assay parameters. (*A*) Tail% (*r* = 0.294, *p* = 0.00). (*B*) Tail length (*r* = 0.194, *p* = 0.005). (*C*) TDM (*r* = 0.204, *p* = 0.00).

As shown in Table 3 and Figure 1, the tail% increased, on average, by 13.26% [95% confidence interval (CI), 7.97–18.55] per 1-unit increase in the log_10_ of urinary 2-OHNa (micrograms per gram creatinine). Log_10_-transformed 2-OHNa was also significantly associated with tail length and TDM (12.25; 95% CI, 0.01–24.52 and 7.55; 95% CI, 1.28–18.83, respectively). Additionally, a positive relationship was found between 1-OHP and tail% (5.32; 95% CI, 0.47–10.17). Log_10_-transformed 2-OHFlu was weakly associated with tail% (5.04; 95% CI, −0.99 to 11.07; *p* = 0.07).

We also summed the total of four PAH metabolites (2-OHNa, 2-OHFlu, 9-OHPh, and 1-OHP) and found that total PAH associated with the increased tail% (15.96; 95% CI, 8.86–23.07). However, there were still no associations between the total PAH metabolites and semen parameters (data not shown).

## Discussion

Chongqing is a heavily industrialized city and suffers from significant air pollution. Although air conditions have improved recently, air quality is reportedly worse, and the PAH levels are higher in December in this city (CEPB 2007). Additionally, several reports have indicated that sperm quality is subject to seasonal changes because of temperature changes, the length of daylight, or ejaculatory frequency (Carlsen et al. 2004; Chia et al. 2001; Hansen et al. 2010). The winter season in Chongqing sees mild weather with overcast conditions and average temperatures around 10°C. Our previous study of semen quality, which included several months representative of summer, autumn, and winter, also found that season significant affected most of the semen parameters (Li et al. 2009). Therefore, we chose December as our sample time, when levels of PAH pollution are heavy and steady and the climate may have a slight influence on semen quality.

PAHs are a large family of environmental pollutants, and different PAHs may have different toxicological properties (Li et al. 2008). Although 1-OHP is the most commonly used indicator of PAH exposure in many previously reported studies, it may not represent the numerous PAH metabolites. To obtain a more accurate measure of exposure to different compounds, we selected 2-OHNa, 2-OHFlu, 9-OHPh, and 1-OHP as exposure biomarkers to represent different parent compounds. Our results indicated that only 2-OHNa and 2-OHFlu, not 9-OHPh, were associated with sperm DNA damage in the study population in Chongqing.

In this study, detection rates of the four metabolites were similar to another study from Nanjing, one of the biggest cities in eastern China (Xia et al. 2009), but our rates were slightly higher than those found in an American population (Li et al. 2008). We speculate that these variations result from the increase in pollution in China due to the rapid expansion of industry and increase in automobile use. Compared with the Nanjing study, the level of urinary 2-OHNa found in our study was 1.6 times higher, the level of 2-OHFlu was comparable, and the level of 1-OHP was 1.7 times lower. Differences in PAH quantities and composition may result from the different industrial structures and the variance in geography and climate between the two cities. Although there were some differences in the absolute levels of PAH metabolites detected in our study and those of others, there was a similar trend with regard to the relative metabolite levels; 2-OHNa was the PAH metabolite with the highest concentration in all three studies, followed by 2-OHFlu and 1-OHP (Xia et al. 2009; Li et al. 2008).

Experimental studies in rats indicate that PAH exposure is negatively correlated with daily sperm production and sperm motility (Ramesh et al. 2008). Human studies in infertile populations (Xia et al. 2009) suggest a negative association between abnormal semen quality and 1-OHP levels. However, our study found no associations between urinary PAH metabolites and semen parameters. Similarly, in a study in the general population, Rubes et al. (2005) reported that PAH exposures do not change semen quality. One potential explanation for these differences may be the variations in the PAH background levels (the level of urinary 1-OHP in our study was 1.7 times lower than that of the men from the study by Xia et al. 2009). Another reason may be that our study and that by Rubes et al. (2005) were based on general populations compared with the infertile population in the study by Xia et al. (2009)

Apoptotic markers have been considered useful indicators of male fertility (Varum et al. 2007). Several studies have shown that the Annexin V–negative sperm have superior quality compared with vital Annexin V–positive sperm (Hoogendijk et al. 2009; Sion et al. 2004). PAHs may reach the epididymis and then interfere with epididymal function, leading to apoptosis of spermatid (Inyang et al. 2003; Ramesh et al. 2008). We noted that the levels of urinary PAH metabolites were correlated only with PI^+^ cells, not Annexin V^+^/PI^−^ spermatozoa. This phenomenon may occur because mature sperm lack normal apoptotic machinery. The sperm with positive staining for Annexin V may have originated from the apoptotic process that proceeds during spermatogenesis. Those sperm whose apoptosis has started in spermatogenesis may have an increased sensitivity to external damaging agents (Lachaud et al. 2004). The active metabolites of PAH may lead to defects that indirectly induce cell death in fragile and damaged mature sperm.

Our data indicated that exposure to PAHs was associated with sperm DNA damage. Consistent with an earlier study (Meeker et al. 2007), we observed that increased urinary 2-OHNa levels were associated with increased tail%. Reactive metabolites of PAHs might reach the testes and epididymis and then react with sperm DNA to form adducts, causing DNA damage (Gaspari et al. 2003). Additionally, compounds resulting from the oxidation of PAHs have the ability to enter redox cycles, which increased the formation of reactive oxygen species (ROS) (Farmer et al. 2003) and thus caused sperm DNA damage (Barroso et al. 2000). Rather than using the neutral comet assay, which measures only double- strand DNA breaks (Duty et al. 2003b; Meeker et al. 2008), we used the alkaline comet assay in this study to determine the various sperm DNA damages caused by PAHs, that is, base-free sites, single- and double-stranded DNA damages (Sakkas and Alvarez 2010).

DNA damage occurring in the absence of other changes in semen quality is also possible. The presence of defective spermatozoa containing DNA damage does not affect the sperm count and morphology (Agarwal and Said 2003; Sakkas and Alvarez 2010). A growing body of evidence also supports the notion that sperm DNA damage is an objective and independent marker of sperm function. Sperm DNA damage in the male germ line is a major contributor to infertility and is linked to an increased incidence of miscarriage and the appearance of various kinds of birth defects in the offspring (Aitken et al. 2009; Fernández-Gonzalez et al. 2008; Morris et al. 2002).

Smoking is reported as a risk factor for male reproductive function and may confound the analyses for the relationships between environmental pollution and sperm quality (Calogero et al. 2009). In our study, after adjusting for smoking status, we found that the coefficients differed < 12% [see Supplemental Material, Tables 1 and 2 (doi:10.1289/ehp.1002340)]. That result seemingly indicates that cigarette smoking changed the association between environmental PAH exposures and sperm DNA damage slightly, which appears to support the findings of other studies (Gammon et al. 2002; Rybicki et al. 2008) that cigarette smoking and other lifestyle habits slightly modulate the risk of environmental PAH exposures.

Crude and adjusted regression coefficients were similar after adjusting for grilled and smoked food ingestion, which is considered an antecedent of PAHs [see Supplemental Material, Table 3 (doi:10.1289/ehp.1002340)]. These results indicate that the potential confounding effect was minimal. However, because of different dietary habits, variation in personal characteristics of volunteers, and cooking variation, it is difficult to ascertain the levels of PAH in food by questionnaire in environmental epidemiological studies. Therefore, dietary sources of PAH exposure need to be fully considered in future studies with an improved study design. In addition, our results should be interpreted with caution because the PAH metabolite levels observed in our study may be associated with other potential sources of PAHs, such as passive smoking (Suwan-ampai et al. 2009). Because there are no strict smoking bans in public places in China, we were not able to carry out a quantitative analysis for passive smoking. Nevertheless, a better measure for passive smoking is needed in future studies.

In our study, we employed urinary PAH metabolites as biomarkers of PAH exposure rather than the conventional methods for monitoring environmental pollution, because biomarkers have the potential to integrate exposure to chemicals from all sources and routes of exposure (Duty et al. 2003a). However, a limitation of using biomarkers is that they usually do not allow for determination of primary exposure sources and different analysis of the parent compounds (Meeker et al. 2008). Additionally, the time interval for spermatogenesis is about 90 days, longer than the half-life of PAH metabolites (several hours to days). Using only a single urine sample to predict metabolite concentrations over longer periods may be a potential limitation of our study. Although chronic repeated exposure to exogamic material may result in a steady-state level of PAH metabolites and thus improved accuracy in estimating exposure using urinary metabolites, the temporal variability in metabolites from environmental exposure might have led to a bias during our study. It is possible that participants may have had different exposures on different days based on their occupation, travel, or other factors. Thus, although we believe that urinary metabolites provide an initial estimate of exposure, the use of additional biomarkers (e.g., DNA adduct formation) that can accurately reflect chronic exposure should be considered in future studies.

## Conclusion

The environmental exposure levels of some PAHs affect human spermatozoa quality and male fertility. Our study indicates that PAHs may disrupt male reproduction by damaging sperm DNA, rather than disrupting semen quality and triggering apoptosis of mature sperm. We speculate that the potential impact of exposure to environmental pollutants on human sperm DNA should be considered. Future large-scale studies should incorporate different markers and different seasons to generate a more accurate and full assessment of the adverse effects of PAH exposure on male fertility.

## Figures and Tables

**Table 1:** Characteristics of the study participants (*n* = 232).

Characteristic	Value
Age (years)	31.89 ± 5.53
BMI (kg/m^2^)	22.55 ± 2.76
Abstinence duration (days)	4.63 ± 1.77
Tobacco use (cigarettes/day)	
None	87 (37.50)
≤ 10	71 (30.60)
> 10	74 (31.90)
Alcohol use (grams/month)	
None	75 (32.30)
≤ 120	139 (59.90)
> 120	18 (7.80)
Grilled/smoked food consumption (grams/week)	183.78 ± 137.73

Values are mean ± SD or no. (%).

**Table 2:** Distribution of PAH metabolites and sperm quality parameters.

Characteristic	Geometric mean	Selected percentiles		
		5th	50th	95th
PAH metabolite (μg/g creatinine)				
2-OHNa	7.72	1.16	8.77	20.94
2-OHFlu	2.95	1.14	3.25	9.43
9-OHPh	1.92	0.64	2.15	6.57
1-OHP	0.66	0.13	0.64	2.94

Apoptotic marker				
Annexin V^−^/PI^−^ spermatozoa (%)	67.46	40.22	73.22	88.55
Annexin V^+^/PI^−^ spermatozoa (%)	6.25	2.49	7.11	16.58
PI^+^ spermatozoa (%)	17.20	5.75	8.11	53.36

Comet parameter				
Tail%	32.24	1.67	35.91	61.08
Tail length (μm)	77.11	18.48	90.60	128.61
TDM (μm)	29.24	11.51	30.76	60.21

Abbreviations: V^−^/PI^−^, living cells without PS translocation; V^+^/PI^−^, living cells with PS translocation; PI^+^, necrotic cells.

**Table 3:** Adjusted regression coefficients^a^ (95% CI) of urinary PAH metabolites and annexin V/PI and comet assay parameters.

Outcome	2-OHNa^b^	2-OHFlu^b^	9-OHPh^b^	1-OHP^b^	∑PAH metabolites^c^
Annexin V/PI parameter					
Annexin V^−^/PI^−^ (%)	−9.59 (−15.78 to −3.41)**	−11.10 (−17.31 to −4.88)**	−7.37 (−13.04 to −1.69)**	−5.78 (−11.17 to 0.37)*	−13.02 (−21.55 to −4.50)**
Annexin V^+^/PI^−^ (%)^b^	−0.02 (−0.12 to 0.09)	−0.01 (−0.11 to 0.09)	−0.01 (−0.10 to −0.08)	0.02 (−0.07 to 0.10)	0.03 (−0.11 to 0.17)
PI^+^ (%)	9.68 (3.58 to 15.72)**	8.91 (2.99 to 14.84)**	6.69 (1.32 to 12.05)**	4.56 (−0.56 to 9.68)*	12.59 (4.15 to 21.02)**

Comet parameter					
Tail%	13.26 (7.97 to 18.55)**	5.04 (−0.99 to 11.07)*	3.32 (−1.97 to 8.62)	5.32 (0.47 to 10.17)**	15.96 (8.86 to 23.07)**
Tail length	12.25 (0.01 to 24.52)**	6.48 (−6.30 to 19.26)	5.23 (−5.98 to 16.43)	2.60 (−8.05 to 13.24)	16.56 (−0.39 to 33.52)*
TDM	7.55 (1.28 to 18.83)**	3.39 (−3.08 to 9.86)	3.58 (−2.00 to 9.14)	1.75 (−3.55 to 7.05)	6.29 (−2.36 to 14.95)

a Regression coefficients were adjusted for age, abstinence, and smoking status.

b Log_10_-transformed.

c ∑PAH metabolites: combination of four PAH metabolites.

** *p* < 0.05.

* *p* < 0.1.

## References

[b1-ehp-119-652] Agarwal A, Said TM (2003). Role of sperm chromatin abnormalities and DNA damage in male infertility. Hum Reprod Update.

[b2-ehp-119-652] Aitken RJ, Iuliis GN, McLachlan RI (2009). Biological and clinical significance of DNA damage in the male germ line. Int J Androl.

[b3-ehp-119-652] Armstrong B, Hutchinson E, Unwin J, Fletcher T (2004). Lung cancer risk after exposure to polycyclic aromatic hydrocarbons: a review and meta-analysis. Environ Health Perspect.

[b4-ehp-119-652] ATSDR (Agency for Toxic Substances and Disease Registry) (1995). Toxicological Profile for Polycyclic Aromatic Hydrocarbons (PAHs).

[b5-ehp-119-652] Barroso G, Morshedi M, Oehringer S (2000). Analysis of DNA fragmentation, plasma membrane translocation of phosphatidylserine and oxidative stress in human spermatozoa. Hum Reprod.

[b6-ehp-119-652] Calogero A, Polosa R, Perdichizzi A, Guarino F, La Vignera S, Scarfia A (2009). Cigarette smoke extract immobilizes human spermatozoa and induces sperm apoptosis. Reprod Biomed Online.

[b7-ehp-119-652] Carlsen E, Petersen J, Andersson AM, Skakkebaek NE (2004). Effects of ejaculatory frequency and season on variations in semen quality. Fertil Steril.

[b8-ehp-119-652] CEPB (Chongqing Environmental Protection Bureau) (2007). Chongqing municipality state of the environment.

[b9-ehp-119-652] Chen Z, Toth T, Godfrey-Bailey L, Mercedat N, Schiff I, Hauser R (2003). Seasonal variation and age-related changes in human semen parameters. J Androl.

[b10-ehp-119-652] Chia SE, Lim ST, Ho LM, Tay SK (2001). Monthly variation in human semen quality in male partners of infertile women in the tropics. Hum Reprod.

[b11-ehp-119-652] Comet Assay Software Project Lab (2004). CaspLab 1.2.2.

[b12-ehp-119-652] Donnelly ET, Steele EK, McClure N, Lewis SE (2001). Assessment of DNA integrity and morphology of ejaculated spermatozoa from fertile and infertile men before and after cryopreservation. Hum Reprod.

[b13-ehp-119-652] Duty SM, Silva MJ, Barr DB, Brock JW, Ryan L, Chen Z (2003a). Phthalate exposure and human semen parameters. Epidemiology.

[b14-ehp-119-652] Duty SM, Singh NP, Silva MJ, Barr DB, Brock JW, Ryan L (2003b). The relationship between environmental exposures to phthalates and DNA damage in human sperm using the neutral comet assay. Environ Health Perspect.

[b15-ehp-119-652] Farmer PB, Singh R, Kaur B, Sramb RJ, Binkova B, Wasilewska AC (2003). Molecular epidemiology studies of carcinogenic environmental pollutants: effects of polycyclic aromatic hydrocarbons (PAHs) in environmental pollution on exogenous and oxidative DNA damage. Mutat Res.

[b16-ehp-119-652] Fernández-Gonzalez R, Moreira PN, Pérez-Crespo M, Sánchez-Martín M, Ramirez MA, Pericuesta E (2008). Long-term effects of mouse intracytoplasmic sperm injection with DNA fragmented sperm on health and behavior of adult offspring. Biol Reprod.

[b17-ehp-119-652] Gammon MD, Santella RM, Neugut AI, Eng SM, Teitelbaum SL, Paykin A (2002). Environmental toxins and breast cancer on Long Island. I. polycyclic aromatic hydrocarbon DNA adduct. Cancer Epidemiol Biomark Prev.

[b18-ehp-119-652] Gaspari L, Chang SS, Santella RM, Garte S (2003). Polycyclic aromatic hydrocarbon-DNA adducts in human sperm as a marker of DNA damage and infertility. Mutat Res.

[b19-ehp-119-652] Greenland S (1989). Modeling and variable selection in epidemiologic analysis. Am J Public Health.

[b20-ehp-119-652] Guo Z, Luo C, Zhang W, Lu Y, Sun J, Cao J (2006). The analysis of the persistent organic pollution in the Three Gorges Reservoir in Chongqing [in Chinese]. Environ Monit.

[b21-ehp-119-652] Hansen C, Luben TJ, Sacks JD, Olshan A, Jeffay S, Perreault SD (2010). The effect of ambient air pollution on sperm quality. Environ Health Perspect.

[b22-ehp-119-652] Hoogendijk CF, Kruger TF, Bouic PJ, Henkel RR (2009). A novel approach for the selection of human sperm using annexin V-binding and flow cytometry. Fertil Steril.

[b23-ehp-119-652] Hsu PC, Chen IY, Pan CH, Wu KY, Pan MH, Chen JR (2006). Sperm DNA damage correlates with polycyclic aromatic hydrocarbons biomarker in coke-oven workers. Int Arch Occup Environ Health.

[b24-ehp-119-652] Inyang F, Ramesh A, Kopsombut P, Niaz MS, Hood DB, Nyanda AN (2003). Disruption of testicular steroidogenesis and epididymal function by inhaled benzo(*a*)pyrene. Reprod Toxicol.

[b25-ehp-119-652] Irvine DS, Twigg JP, Gordon EL, Fulton N, Milne PA, Aitken RJ (2000). DNA integrity in human spermatozoa: relationship with semen quality. J Androl.

[b26-ehp-119-652] Kummera V, Mašková J, Zralý Z, Neča J, Šimečková P, Vondráček J (2008). Estrogenic activity of environmental polycyclic aromatic hydrocarbons in uterus of immature Wistar rats. Toxicol Lett.

[b27-ehp-119-652] Lachaud C, Tesarik J, Cañadas ML, Mendoza C (2004). Apoptosis and necrosis in human ejaculated spermatozoa. Hum Reprod.

[b28-ehp-119-652] Levine RJ, Mathew RM, Chenault CB, Brown MH, Hurtt ME, Bentley KS (1990). Differences in the quality of semen in outdoor workers during summer and winter. New Engl J Med.

[b29-ehp-119-652] Li YF, Lin H, Ma MF, Li LB, Cai M, Zhou N (2009). Semen quality of 1346 healthy men, results from the Chongqing area of southwest China. Hum Reprod.

[b30-ehp-119-652] Li Z, Sandau CD, Romanoff LC, Caudill SP, Sjodin A, Needham LL (2008). Concentration and profile of 22 urinary polycyclic aromatic hydrocarbon metabolites in the U.S. population. Environ Res.

[b31-ehp-119-652] Luben TJ, Olshan AF, Herring AH, Jeffay S, Strader L, Perreault SD (2007). The Healthy Men Study: an evaluation of exposure to disinfection by-products in tap water and sperm quality. Environ Health Perspect.

[b32-ehp-119-652] MacKenzie KM, Angevine DM (1981). Infertility in mice exposed in utero to benzo(a)pyrene. Biol Reprod.

[b33-ehp-119-652] Meeker JD, Barr DB, Hauser R (2008). Human semen quality and sperm DNA damage in relation to urinary metabolites of pyrethroid insecticides. Hum Reprod.

[b34-ehp-119-652] Meeker JD, Barr DB, Serdar B, Rappaport SM, Hauser R (2007). Utility of urinary 1-naphthol and 2-naphthol levels to assess environmental carbaryl and naphthalene exposure in an epidemiology study. J Expo Sci Environ Epidemiol.

[b35-ehp-119-652] Morris ID, Ilott S, Dixon L, Brison DR (2002). The spectrum of DNA damage in human sperm assessed by single cell gel electrophoresis (comet assay) and its relationship to fertilization and embryo development. Hum Reprod.

[b36-ehp-119-652] Payne A (1958). The pathological effects of the intraperitoneal injection of 3:4-benzpyrene into rats and mice. Br J Cancer.

[b37-ehp-119-652] Ramesh A, Inyang F, Lunstra DD, Niaz MS, Kopsombut P, Jones KM (2008). Alteration of fertility endpoints in adult male F-344 rats by subchronic exposure to inhaled benzo(a)pyrene. Exp Toxicol Pathol.

[b38-ehp-119-652] Rubes J, Selevan SG, Evenson DP, Zudova D, Vozdova M, Perreault SD (2005). Episodic air pollution is associated with increased DNA fragmentation in human sperm without other changes in semen quality. Hum Reprod.

[b39-ehp-119-652] Rybicki BA, Neslund-Dudas C, Bock CH, Rundle A, Savera AT, Yang JJ (2008). Polycyclic aromatic hydrocarbon—DNA adducts in prostate and biochemical recurrence after prostatectomy. Clin Cancer Res.

[b40-ehp-119-652] Sakkas D, Alvarez JG (2010). Sperm DNA fragmentation: mechanisms of origin, impact on reproductive outcome, and analysis. Fertil Steril.

[b41-ehp-119-652] Shu WQ, Tian HJ, Cao J (2002). Mutagenic activities and their seasonal changes of organic extracts from source water of Yangtze River and Jialing River in Chongqing section. Environ Pollut Prev.

[b42-ehp-119-652] Sion B, Janny L, Boucher D, Grizard G (2004). Annexin V binding to plasma membrane predicts the quality of human cryopreserved spermatozoa. Int J Androl.

[b43-ehp-119-652] Suwan-ampai P, Navas-Acien A, Strickland PT, Agnew J (2009). Involuntary tobacco smoke exposure and urinary levels of polycyclic aromatic hydrocarbons in the United States, 1999 to 2002. Cancer Epidemiol Biomarkers Prev.

[b44-ehp-119-652] Tian HJ, Shu WQ, Zhang XK (2003). Organic pollutants in source water in Jialing River and Yangtze River (Chongqing section) [in Chinese]. Resour Environ Yangtze Basin.

[b45-ehp-119-652] Varum S, Bento C, Sousa AP, Gomes-Santos CS, Henriques P, Almeida-Santos T (2007). Characterization of human sperm populations using conventional parameters, surface ubiquitination, and apoptotic markers. Fertil Steril.

[b46-ehp-119-652] Vermes I, Haanen C, Steffens-Nakken H, Reutelingsperger C (1995). A novel assay for apoptosis. Flow cytometric detection of phosphatidylserine expression on early apoptotic cells using fluorescein labelled Annexin V. J Immunol Methods.

[b47-ehp-119-652] Vinggaard AM, Hnida C, Larsen JC (2000). Environmental polycyclic aromatic hydrocarbons affect androgen receptor activation *in vitro*. Toxicology.

[b48-ehp-119-652] Wang Y, Zhang WB, Dong YL, Fan RF, Sheng GY, Fu JM (2005). Quantification of several monohydroxylated metabolites of polycyclic aromatic hydrocarbons in urine by high-performance liquid chromatography with fluorescence detection. Anal Bioanal Chem.

[b49-ehp-119-652] WHO (World Health Organization) (1999). WHO Laboratory Manual for the Examination of Human Semen and Semen–Cervical Mucus Interaction.

[b50-ehp-119-652] Wyrobek AJ, Eskenazi B, Young S, Arnheim N, Pearson FS, Evenson D (2006). Advancing age has differential effects on DNA damage, chromatin integrity, gene mutations, and aneuploidies in sperm. Proc Natl Acad Sci USA.

[b51-ehp-119-652] Xia YK, Zhu PF, Han Y, Lu CC, Wang SL, Gu A (2009). Urinary metabolites of polycyclic aromatic hydrocarbons in relation to idiopathic male infertility. Hum Reprod.

